# Editorial: Adaptation of Dryland Plants to a Changing Environment

**DOI:** 10.3389/fpls.2019.01228

**Published:** 2019-10-08

**Authors:** Zhiyou Yuan, Yinglong Chen, Jairo A. Palta, P. V. Vara Prasad

**Affiliations:** ^1^State Key Laboratory of Soil Erosion and Dryland Farming on the Loess Plateau, Northwest A&F University, Yangling, China; ^2^State Key Laboratory of Soil Erosion and Dryland Farming on the Loess Plateau, Institute of Soil and Water Conservation, Chinese Academy of Sciences and Ministry of Water Resources, Yangling, China; ^3^The UWA Institute of Agriculture and School of Agriculture and Environment, The University of Western Australia, Perth, WA, Australia; ^4^CSIRO Agriculture & Food, Wembley, WA, Australia; ^5^Department of Agronomy, Kansas State University, Manhattan, KS, United States

**Keywords:** future climates, drylands, ecosystem functioning, global change, plant adaptation, vegetation dynamics

Currently, the earth is undergoing rapid and major environmental changes mostly because of anthropogenic activities (IPCC, 2014). The biogeochemical cycles and the structure and function of ecosystems in several regions of the world are being substantially changed due to water scarcity and climate change (Koutroulis, 2019). The dryland ecosystem, which covers 40−50% of earth’s terrestrial surface and is home to more than a third of the world’s human population (Mortimore et al., 2009; Schimel, 2010), is being dramatically affected the most. The changes in the environment are not only amplifying the pressure exerted on the drylands but also placing new challenges (Gonzalez-Megias and Menendez, 2012; Reed et al., 2012; Koutroulis, 2019). The effect of such human-caused environmental changes is already recognizable in the responses and dynamics of dryland ecosystems (e.g., Maestre et al., 2015; Butterfield and Munson, 2016; Schlaepfer et al., 2017). The adaptation of dryland plants and their development, fitness, and competitiveness in a changing environment, however, are currently poorly understood. There is no doubt that scientists need to improve their understanding of the mechanisms of plant adaptation and the processes that are triggered by human-driven changes to the environment and provide that information to policy makers and dryland managers to develop strategies for sustainable management of dryland ecosystems. The studies included in this research topic examined how dryland plants respond and evolve in a changing environment. Addressing questions on the impact of environmental change on drylands, with a particular focus on drought (limited water) and heat (high temperature), not only offers new perspectives but also provides ideas and outlines critical challenges that need to be researched.

Typically, the ecological understanding of the response to environmental conditions and changes rely on three approaches: (i) manipulative experiments, (ii) long-term observational records by monitoring the response to ambient environmental fluctuations using repeat sampling of plots, and (iii) space-for-time substitutions derived from sampling along environmental gradients and across time scales. The three approaches are valuable but sometimes produce inconsistent estimates of the magnitude of plant community response to environmental changes (Yuan et al., 2017; Barner et al., 2018; Knapp et al., 2018). There is therefore a need for using a combination of experimental, monitoring, and gradient approaches to provide different insights on the response to environmental changes. This research topic brings together the results from a number of studies exploring plant adaptation to a changing environment in drylands using such different methods.

Six of the 12 articles in the issue deal with manipulative experiments, an ongoing commonly used approach in ecology research. They address a variety of topics, notably the responses of a vast range of dryland plant species to experimental drought (Mahajan et al.; Wang et al; Li et al.) and nutrient addition (Cui et al.). Mahajan et al. quantified the impact of moisture stress on the physiological changes and reproductive capacity of emerging problematic weed species (*Sisymbrium thellungii*) to understand reasons for its spread and develop appropriate management strategies. Wang et al. measured the performance of root systems of two contrasting plant species (*Bothriochloa ischaemum*, C_4_ herbaceous species, and *Lespedeza davurica*, C_3_ leguminous species) under different soil moisture regimes to understand interactions between the shrub–grass species. Cui et al. measured the N response of two temperate desert plant species (*Malcolmia africana*, an ephemeral, and *Salsola affinis*, an annual) to understand their aboveground and belowground growth performance and N retention, thus helping understand the relative competitiveness and species composition under different environmental conditions. Li et al. conducted experiments to understand the interaction between endophytes isolated from desert plants on the performance of *Hedysarum scoparium* under different soil water conditions. They observed that endophytes established a positive symbiosis and further enhanced the biomass and antioxidant activities of the host plant particularly under water-deficit conditions.

The study of Zhou et al. reviewed leaf gas exchange in response to experimental drought in drylands and provides new information on the responses of plant function to drought, highlighting the importance of linking plant traits and particularly the correlation between hydraulic conductivity and photosynthesis. In their study in northern China, Liu et al. describe the effects of defoliation caused by animal grazing and hay production on plant regrowth and conclude that resource reallocation is specific to species. In drylands, particularly in deserts, plants and their seeds often experience various degrees of sand burial and exposure to wind erosion. Plant adaptation to these environmental stresses is thus critical for successful seed germination, seedling emergence, and initial establishment (Pimentel and Kounang, 1998). The study of Fan et al. showed that desert shrubs take a bet-hedging strategy to adapt to such arid environments. All of these studies report significant changes in the processes of dryland plants responding to various experimental treatments, highlighting the importance of even a tiny alteration of environments in drylands for their functioning. Therefore, the studies on this research topic could provide a powerful tool to establish cause–effect relationships and to test specific hypotheses regarding the impacts of environmental change.

In the study of Haberstroh et al. terpenoid emissions from two woody species that have developed photoprotective mechanisms to adapt to environmental pressures associated with Mediterranean climate were monitored. Gang et al. monitored drought conditions in China’s drylands over 12 years and found that the carbon assimilated and the water used by dryland forests were more affected by drought than in dryland grasslands. Yan et al. investigated the possible drought-related patterns of plant functional traits across a macroecological gradient, that is, a precipitation transect where they sampled 39 dominant species in 22 sites in the Inner Mongolian grassland, finding that dryland plants have adapted to drought in four different ways. Fang et al. focused on the variation in plant/soil carbon, nitrogen, and phosphorus stoichiometry along a latitudinal gradient of ∼500 km in northern China’s drylands. Both studies demonstrate that environmental changes are likely to affect plant cover, community composition, and functional traits. The studies also highlight that environmental change can cause significant impacts on drylands. By analyzing 720 soil samples from 72 paired sites in northeastern China, Wu et al. discuss the role of deep soils on dryland plants at a large geographic scale.

The research described in this research topic highlights the various mechanisms underlying the potential response of dryland plants to a changing environment, dependent on the component considered and the amount and duration of the abiotic or biotic stress. The plant-soil-environment feedback, together with other disturbances, might lead to functional alterations of ecosystem resilience in drylands. Short- and long-term adaptations may differ, which is why experiments, monitoring, and gradient observations are needed (Elmendorf et al., 2015; Yuan and Chen, 2015; Blume-Werry et al., 2016; Yuan et al., 2017). In particular, long-term environmental changes could result in a new stable state of the dryland ecosystem, and its structural persistence might strongly depend on the adaptive capacity/plasticity of individuals and populations to environmental impacts. As shown in this special issue, drylands are inhabited by many species that respond in different ways to a changing environment. Undoubtedly, the broad range of articles in this research topic could deepen our current understanding of ecological mechanisms by which plants respond and adapt to environmental changes in dryland ecosystems. This topic still remains as a frontier in plant science with an urgent need to be understood to predict the impacts of environmental changes on drylands.

## Author Contributions

ZY organized the research topic together with YC, JP, and PP. ZY wrote the first draft of the editorial, with all coauthors jointly editing the final version.

## Funding

This work was supported by the National Key Research and Development Program of China (2016YFA0600801) and the National Natural Sciences Foundation of China (31570438) toZY, Chinese Academy of Sciences’ “100 Talent” Program to ZY and YC.

## Conflict of Interest

The authors declare that the research was conducted in the absence of any commercial or financial relationships that could be construed as a potential conflict of interest.
